# Deep Learning-Based Classification of Zirconia and Metal-Supported Porcelain Fixed Restorations on Panoramic Radiographs

**DOI:** 10.3390/diagnostics16070972

**Published:** 2026-03-25

**Authors:** Zeynep Başağaoğlu Demirekin, Turgay Aydoğan, Yunus Cetin

**Affiliations:** 1 Faculty of Dentistry, Suleyman Demirel University, Isparta 32260, Turkey; yns_yns1245@hotmail.com; 2 Faculty of Engineering and Natural Sciences, Suleyman Demirel University, Isparta 32260, Turkey; turgayaydogan@sdu.edu.tr

**Keywords:** artificial intelligence, zirconia-based fixed restorations, metal-supported porcelain veneer

## Abstract

**Background/Objectives:** This study aimed to automatically classify Zirconia-based fixed restorations and porcelain-fused-to-metal (PFM) restorations on panoramic radiographs using an artificial intelligence-based model. Unlike previous studies that mainly focused on classifying types of restorations (e.g., crowns, fillings, implants), this research concentrated on material-based differentiation, aiming to provide a more specific contribution to clinical decision support systems. **Method**: Panoramic radiographs obtained from the archive of Süleyman Demirel University Faculty of Dentistry were included in this study. Radiographs with poor image quality or insufficient visibility of the restoration area were excluded. A total of 593 cropped region-of-interest (ROI) images, labeled by expert prosthodontists using ImageJ software (version 1.54r; National Institutes of Health, Bethesda, MD, USA), were included in the analysis. In order to reduce class imbalance, data augmentation was applied only for images in the Zirconia-based fixed restorations class. By using various image processing techniques such as rotation, reflection and brightness change, the number of samples in the zirconia-based restorations class was increased and thus a balanced dataset was obtained with a close number of samples for both classes. For model training, the pre-trained VGG16 architecture was used with a transfer learning method, and the final layers were retrained and fine-tuned. The model was configured specifically for binary classification. The entire dataset was randomly split into 70% training, 20% validation, and 10% testing. Model performance was evaluated using accuracy, F1-score, sensitivity, and specificity. **Results**: The model correctly classified 90 out of 94 images in the test dataset, achieving an overall accuracy rate of 96%. For both classes, the precision, recall, and F1-score values were measured in the range of 95% to 96%. Additionally, the Area Under the Curve (AUC) of the ROC curve was calculated as 0.994, and the Average Precision (AP) score was determined to be 0.995. According to the confusion matrix results, only 4 images were misclassified, consisting of 2 false positives and 2 false negatives. **Conclusions**: The deep learning model demonstrated high accuracy in differentiating zirconia and metal-supported porcelain restorations on panoramic radiographs, suggesting that material-based AI classification may support clinical decision-making in restorative dentistry.

## 1. Introduction

Radiographic imaging plays a fundamental role in dentistry, serving as an essential tool for diagnosis, treatment planning, and the detection of pathologies that are not visible during clinical examination. Various radiographic modalities, including panoramic, cephalometric, hand-wrist, periapical, occlusal, and bitewing radiographs, are routinely used in clinical practice to support diagnostic decision-making.

Among these modalities, panoramic radiographs are widely preferred due to their broad anatomical coverage. However, their interpretation can be challenging and requires substantial clinical expertise, and even experienced clinicians may occasionally overlook certain findings during evaluation. Recent developments in artificial intelligence, particularly deep learning, have contributed to significant progress in medical image analysis [1,2] and have led to growing interest in dental applications [3]. Although AI-based analysis of dental radiographs remains more limited than in other medical imaging fields, several dental conditions—including missing teeth, crown restorations, dental caries, periodontal diseases, and periapical lesions—have been detected using deep learning models trained on two-dimensional radiographic datasets [4,5,6].

Artificial intelligence systems are capable of performing various image analysis tasks such as object detection, segmentation, and classification [1]. For these tasks to be performed successfully, artificial intelligence models must be trained using image data and corresponding labeled datasets [3]. In addition, achieving reliable performance requires appropriate evaluation metrics and statistical validation [7]. Previous studies have applied deep learning models to panoramic radiographs for the detection of teeth, restorations, and other dental structures. These studies demonstrated that artificial intelligence can identify dental restorations with promising accuracy, supporting its potential role in automated radiographic analysis [4,5,6].

Metal-supported porcelain (porcelain-fused-to-metal, PFM) restorations have been widely used for decades due to their mechanical reliability and long-term clinical performance; however, esthetic limitations and potential biological concerns related to metal substructures have been reported. [8,9]. Zirconia-based restorations may be fabricated in different structural designs. These include monolithic (full-contour) zirconia restorations and zirconia frameworks veneered with ceramic. Monolithic zirconia restorations consist entirely of zirconia and are characterized by high strength and homogeneous radiopacity. In contrast, veneered zirconia restorations include a zirconia substructure covered with a porcelain veneering layer to improve esthetics. These structural differences may influence radiographic appearance due to variations in material composition and thickness. Understanding these differences is important when interpreting radiographic images and may affect the performance of automated classification systems. [9,10,11]. Radiographically, differences in core structure and material composition may influence opacity patterns and X-ray attenuation characteristics [12,13,14]. Distinguishing these materials may therefore be clinically relevant in retreatment planning, marginal evaluation, and in cases where previous treatment records are unavailable [12,13].

These structural and compositional differences suggest that metal-supported and zirconia-based restorations may exhibit distinguishable radiographic patterns due to their material-dependent X-ray attenuation characteristics [11,13,14], providing a rational basis for material-specific classification using deep learning algorithms and underscoring their potential clinical relevance.

Despite these developments, research addressing the differentiation of restorative materials in panoramic radiographs remains limited. Material-based classification may offer additional clinical value because restorative materials can differ in radiographic appearance, treatment requirements, and long-term behavior. Further work in this area may help clarify whether these material-related differences can be distinguished reliably through automated deep learning approaches.

In this study, “zirconia-based fixed restorations” refers to zirconia restorations used as full-contour (monolithic) crowns/bridges and zirconia frameworks veneered with ceramic, whereas “metal-supported porcelain” refers to porcelain-fused-to-metal (PFM) restorations with a metal substructure veneered by porcelain (single crowns and fixed dental prostheses).

Therefore, the present study investigates the feasibility of using a deep learning-based model to classify fixed prosthetic restorations according to their material properties in two-dimensional panoramic radiographs. This study aimed to classify fixed prosthetic restorations according to material composition (zirconia-based vs. metal-supported porcelain) on panoramic radiographs.

The null hypothesis was that a deep learning model would not achieve discriminative performance beyond chance level in differentiating zirconia-based and metal-supported porcelain fixed restorations on panoramic radiographs.

## 2. Materials and Methods

### 2.1. Ethical Approval and Study Design

The study was conducted with ethical approval obtained from the Ethics Committee of Suleyman Demirel University Faculty of Medicine, with its decision dated 13 January 2025 and numbered E.924324. This retrospective image-based study aimed to classify fixed prosthetic restorations on panoramic radiographs according to material category (zirconia-based restorations vs. porcelain-fused-to-metal [PFM] restorations) using a deep learning model.

### 2.2. Sample and Data Collectionlass Balance 

The sample size was calculated using G*Power 3.1.9.7 software with an effect size of 0.3, alpha = 0.05, and power = 0.90, and a minimum of 750 images was determined. Panoramic radiographs acquired between January 2015 and December 2025 were retrospectively screened. All images were consecutively retrieved from the institutional digital archive within this period. No pre-selection based on material type was performed prior to applying the inclusion and exclusion criteria.

All radiographs were digitally obtained using a Planmeca Promax device (Planmeca, Helsinki, Finland) with exposure parameters of 66–68 kVp, 7–13 mA, and 16 s.

A total of 2926 patient images were scanned, and panoramic radiographs from 269 patients were evaluated according to the inclusion and exclusion criteria. Areas containing fixed restorations were identified and cropped to ensure that each restoration was isolated from adjacent structures, and these regions were defined as ROIs (Regions of Interest) with dimensions of 200 × 300 pixels. During both training and testing phases, the model was presented only with cropped ROI images (200 × 300 pixels) containing the isolated restoration area, rather than the entire panoramic radiograph. The ROI determination steps were performed using ImageJ software. As a result, a total of 593 cropped images were obtained, including 426 metal-supported porcelain and 167 zirconia-based fixed restorations. ROI selection and labeling were performed independently by two experienced prosthodontists. Prior to labeling, a calibration session was conducted using 50 randomly selected images. Inter-examiner agreement was assessed using Cohen’s kappa coefficient, which demonstrated excellent agreement (κ = 0.92). In cases of disagreement, consensus was reached through joint evaluation.

**Figure 1:**
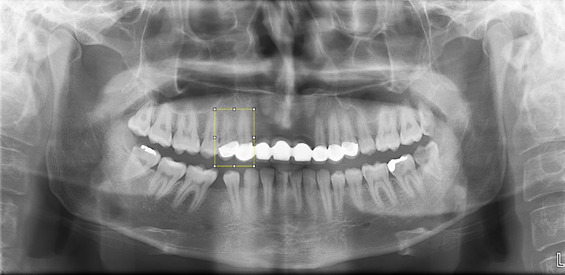
Cropped panoramic radiographs showing zirconia-based fixed restoration and metal-supported porcelain fixed restoration. The yellow box indicates the selected ROI.

**Figure 2:**
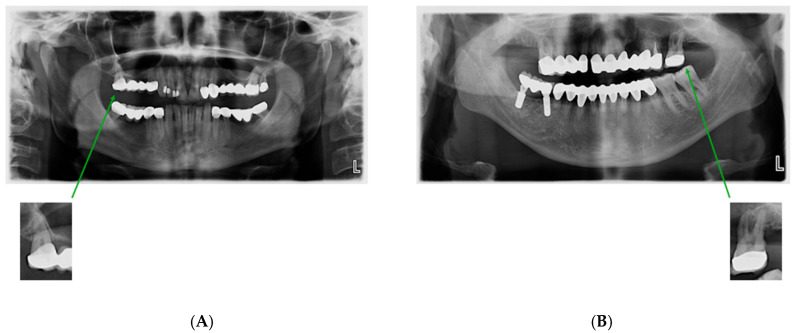
Examples of isolated ROI images: (**A**) zirconia-based restoration exhibiting homogeneous radiopacity; (**B**) metal-supported porcelain restoration showing a dense radiopaque core.

Although both zirconia and metal-supported porcelain restorations exhibit radiopacity on panoramic radiographs, material identification was not based solely on radiographic appearance. Labeling was performed by reviewing corresponding patient treatment records, including laboratory prescriptions and clinical documentation confirming the restorative material used. Radiographic appearance was used only for ROI localization, while final material classification was determined using documented clinical records. This approach minimizes the risk of material misclassification. The cropping procedure is shown in Figure 1 and Figure 2.

**Inclusion criteria were defined as follows:**

• Availability of a post-treatment panoramic radiograph
• The restoration area is clear and distinguishable in the image
• Presence of fixed restoration in the mouth
• Treatment performed at Suleyman Demirel University Faculty of Dentistry

**Exclusion criteria were as follows:**

• Insufficient image quality (blurring, artifacts, positioning errors)
• Restorations on implants, teeth with root canal treatment, or teeth containing only fillings

### 2.3. Data Augmentation

Data augmentation was applied exclusively to the zirconia class within the training set to avoid potential data leakage.

**Figure 3:**
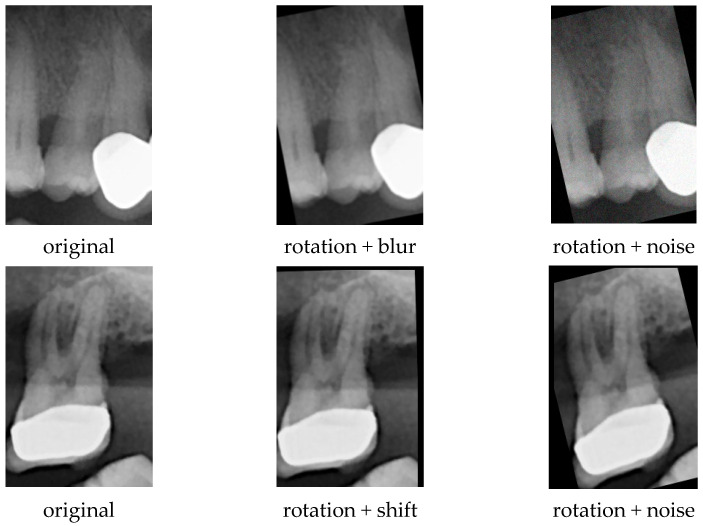
Data Augmentation.

Each of the 167 ROI images was first randomly rotated between −20° and +20°, after which one additional transformation (flip, crop, noise, blur, or shift) was randomly applied. This procedure was performed twice for each image, generating augmented samples. Examples of the augmentation applied to the ROIs are presented in Figure 3. As a result, the class size increased to 501, achieving balance with the metal-supported porcelain class.

After data augmentation, 501 zirconia-based restoration ROI images and 426 PFM restoration ROI images were included and combined into a dataset of 927 samples, which was randomly divided into training, validation, and test subgroups (Table 1). Class balance was maintained within each subgroup, and all images were rescaled to 224 × 224 pixels to ensure compatibility with the VGG16 model.

### 2.4. Deep Learning Model and Fine-Tuning

The data augmentation and model development processes were performed in a Python 3.8 environment using the TensorFlow 2.18.0 and Keras 3.9.0 libraries. The training and testing processes were executed on the Google Colaboratory platform with GPU support, and statistical analyses and data visualizations were performed using the Scikit-learn 1.6.1, NumPy 2.0.2, and Matplotlib libraries 3.10.0.

In this study, the VGG16 (Visual Geometry Group) convolutional neural network, pre-trained on the ImageNet dataset, was utilized and fine-tuned through transfer learning to perform the image classification task. In the proposed model, the initial layers of the VGG16 network were kept frozen, while the last 30 layers were unfrozen and retrained. BatchNormalization layers were left unchanged to preserve their learned normalization parameters. The model was configured for binary classification, distinguishing between Zirconia-based fixed restorations and porcelain-fused-to-metal (PFM) crowns.

**Figure 4:**
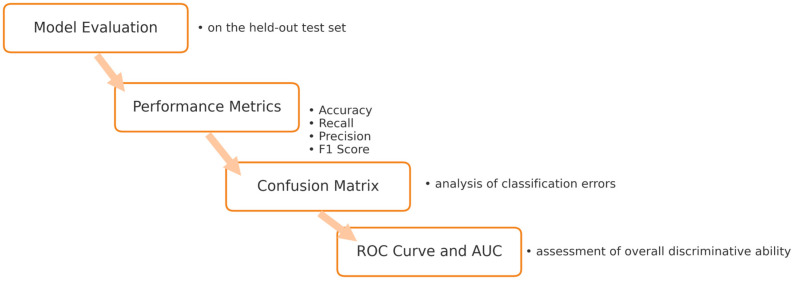
Deep Learning Model.

The model was trained for 50 epochs with a batch size of 32, using the binary cross-entropy loss function and the Adam optimization algorithm (with a learning rate of 1 × 10^−5^). The model’s performance was evaluated using accuracy, recall, precision, F1-score, confusion matrix, and ROC-AUC metrics. The flow diagram of the proposed model is presented in Figure 4.

## 3. Results

In this study, the VGG16 deep learning model was employed to classify zirconia-based fixed restorations and porcelain-fused-to-metal (PFM) restorations in panoramic radiographs. The model’s performance was evaluated using accuracy, recall, precision, F1-score, confusion matrix, and ROC-AUC metrics [15,16,17].

### 3.1. Model Performance and Classification Accuracy

A total of 927 cropped images were used for training, and the model was trained for 50 epochs. During testing, the model’s classification performance was evaluated on 94 test images that were not included in the training or validation sets. In the two-class classification task, when the metal-supported porcelain class was considered positive, the number of true positives (TP) was 41, true negatives (TN) were 49, and false positives (FP) and false negatives (FN) were two each (Table 2). When the Zirconia-based fixed restorations class was considered positive, these values changed symmetrically (Table 3). These results indicated that the model achieved high and balanced accuracy for both classes.

### 3.2. Confusion Matrix

**Figure 5:**
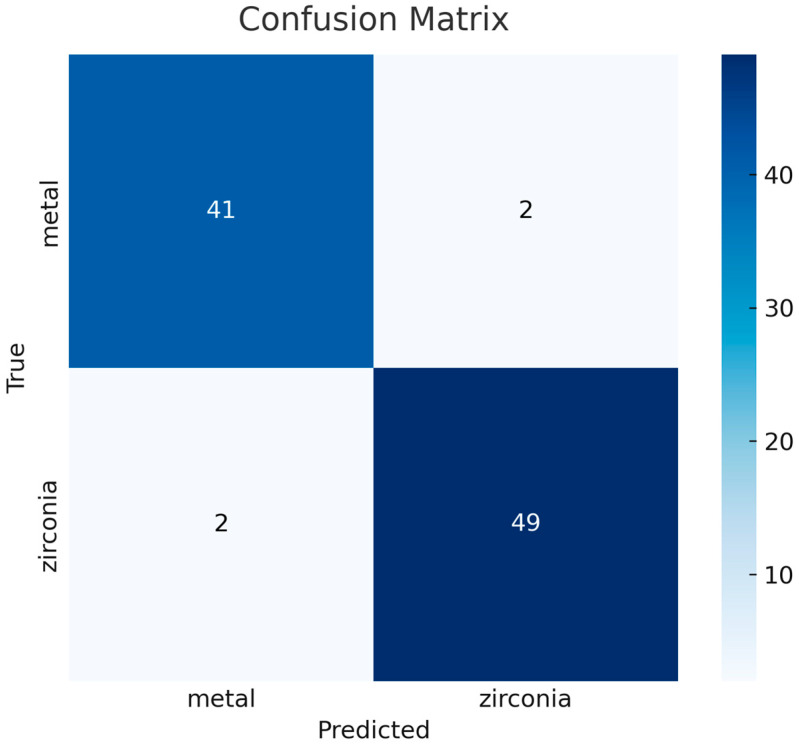
Confusion Matrix.

The confusion matrix illustrates the agreement between the model’s predictions and the ground-truth labels. The model correctly classified 41 of 43 metal-supported porcelain cases and 49 of 51 zirconia-based cases. A low error rate of approximately 4% was observed in the test set, indicating that the model accurately distinguished between the two restoration types (Figure 5).

### 3.3. TPR and FPR Values

True Positive Rate (TPR) and False Positive Rate (FPR) values were calculated during model evaluation. TPR represents the proportion of correctly identified positive samples, whereas FPR represents the rate at which negative samples were misclassified as positives. The TPR and FPR values for both classes indicate strong discriminative performance, with high sensitivity and low false-positive rates (Equations (1)–(4)).

(1) $$TPR=\frac{TP}{TP+FN}$$

TPR Formula [11].

(2) $$FPR=\frac{FP}{FP+TN}$$

Formula for FPR [11].

(3) $$TPR=Recall=\frac{TP}{TP+FN}=0.95\;FPR=\frac{FP}{FP+TN}=\frac{2}{2+49}=\frac{2}{51}\approx 0.039$$

Calculation of TPR and FPR Values for the Porcelain-fused-to-metal (PFM) Class.

(4) $$TPR=Recall=0.96\;FPR=\frac{2}{2+41}=\frac{2}{43}\approx 0.0465$$

Calculation of TPR and FPR Values for the Zirconia-based fixed restorations Class.

### 3.4. Precision, Recall, and F1 Score

Precision, recall, and F1 score achieved values of 95% for the metal-supported porcelain class and 96% for the Zirconia-based fixed restorations class (Table 4). These results showed that the model distinguishes between the two restoration types with balanced performance across classes. The formulas used to obtain these results are shown in Equations (5)–(7).

(5) $$Precision=\frac{TP}{TP+FP}$$

Calculation of Precision Value [12].

(6) $$Recall=\frac{TP}{TP+FN}$$

Calculation of Recall Value [12].

(7) $$F1\;Score=2\times \frac{Precision\times Recall}{Precision+Recall}$$

Calculation of the F1 Score.

### 3.5. ROC Curve and AUC

**Figure 6:**
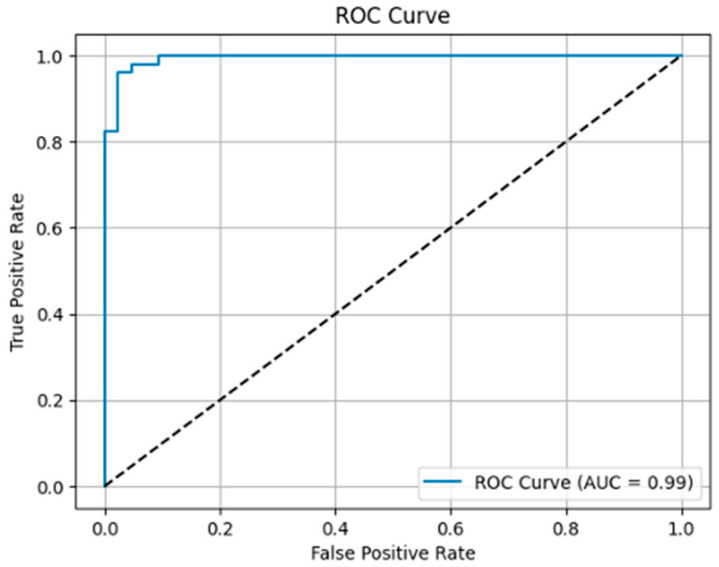
ROC Curve.

The ROC curve visually presents the model’s ability to distinguish between positive and negative classes. The model’s AUC value was calculated as 0.99, indicating nearly perfect discrimination power (Figure 6).

### 3.6. Precision-Recall (PR) Curve

**Figure 7:**
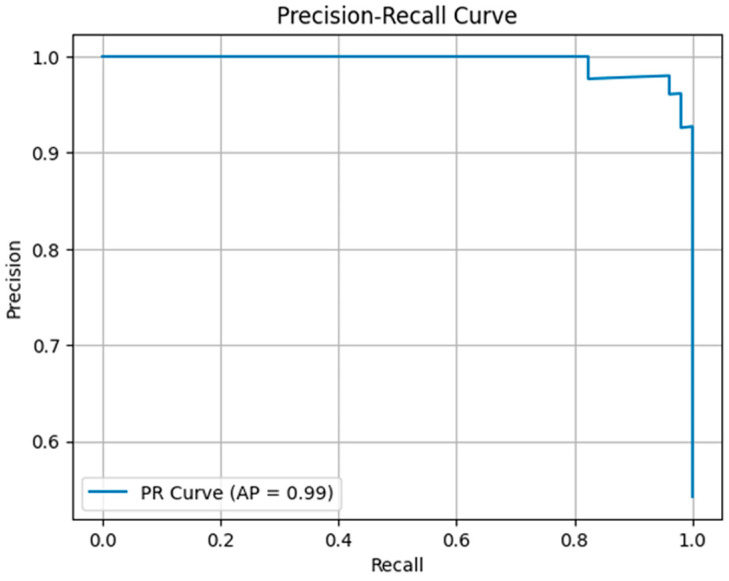
Precision-Recall Curve.

The PR curve was used to evaluate the performance of the positive class specifically. The model’s Average Precision (AP) achieved a value of 0.99, indicating that the model accurately identifies positive samples with high reliability. The PR curve provides a more sensitive evaluation of model performance, especially in datasets with class imbalance (Figure 7).

## 4. Discussion

Considering that the literature generally focuses on identifying the presence or general types of restorations (e.g., crowns, fillings, implants), this study aimed to contribute to the field by classifying restorations based specifically on material properties. The null hypothesis (H_0_), which stated that the artificial intelligence model would not distinguish between zirconia and porcelain-fused-to-metal (PFM) restorations above chance level, was clearly rejected. The model achieved 96% overall accuracy, with F1-scores of 0.96 for zirconia and 0.95 for metal-supported porcelain restorations, demonstrating strong discriminative performance.

In dentistry, artificial intelligence has been increasingly integrated into diagnostic processes, data management, and workflow automation, thereby supporting personalized and predictive dental care [18,19]. Trained using human expertise, AI models serve as valuable tools that aid clinicians across multiple stages of diagnosis and decision-making [20,21,22,23]. Previous studies have shown the effectiveness of AI in periapical and bite-wing radiographs for tasks such as tooth detection [24], caries detection [25,26,27,28], anatomical landmark identification [20], and restoration detection [29,30,31,32]. Pauwels et al. [31] demonstrated that AI surpassed radiologists in detecting apical lesions, while Cantu et al. [33] reported higher accuracy for AI models compared to clinicians in caries detection. Similarly, Cha et al. [34] found that AI achieved performance comparable to dentists when evaluating peri-implant bone loss. These findings collectively highlight the capacity of AI to enhance clinical workflows by reliably identifying anatomical structures, pathologies, and restorative materials.

Although numerous studies have examined automated detection and classification of dental restorations in panoramic radiographs [7,35], the majority have remained limited to type-based classification rather than focusing on material differences. Radiographic differentiation between restorative materials is influenced by atomic number, density, and material thickness, which affect X-ray attenuation and image contrast [12,15,16]. Previous radiographic studies have demonstrated measurable differences in opacity between metal alloys and zirconia ceramics, supporting the biological plausibility of material-based image discrimination. This radiographic distinction provides a theoretical foundation for deep learning models to identify subtle material-dependent imaging patterns [12,16].

Abdalla-Aslan et al. [7] reported 93.6% accuracy for restoration classification, and achieved an F1-score of 0.91 for crown detection using Faster R-CNN. Vinayahalingam et al. [35] obtained an F1-score of 0.94 for crown detection in 2000 panoramic radiographs, while Yurttaş et al. [36] achieved an F1-score of 0.99 for crowns, implants, and pontics using YOLOv5x. However, these studies did not differentiate restorations according to material composition. Bonfanti-Gris et al. [37] reported low sensitivity (AUC = 0.672) for resin-based restorations using commercial Denti.AI software, with higher performance observed for radiopaque materials. In contrast, the present study exclusively targeted fixed restorations and successfully differentiated zirconia-based fixed restorations from porcelain-fused-to-metal (PFM)s based on material characteristics, achieving high accuracy (96%) and strong F1-scores (0.96 and 0.95, respectively). An AUC of 0.99 was obtained for radiopaque restorations, underscoring the model’s strong discriminative capability.

Broader AI research in dental radiography also supports these findings. Güneç et al. [38] reported that AI outperformed young dentists in detecting periapical lesions, achieving a sensitivity of 97.3%. Altan et al. [39], achieved 74% precision for crown detection using YOLOv4, attributing performance limitations to variations and artifacts in panoramic images. In implant and radiograph classification tasks, Sukegawa et al. [40] and Kats et al. [41] reported high accuracy using VGG16-based models, while Tuzoff et al. [9] demonstrated over 99% accuracy in tooth detection. These studies highlight the reliability of VGG16 in dental image analysis. In line with this evidence, the present study achieved strong performance in material-based classification despite a relatively limited dataset, benefiting from transfer learning capabilities.

Unlike previous studies primarily focusing on restoration detection or type-based classification using full panoramic inputs, the present study adopted an ROI-based material classification approach. This task definition differs conceptually from object detection frameworks (e.g., YOLO, Faster R-CNN), as it targets intrinsic material characteristics rather than anatomical localization.

Although the model demonstrated high performance metrics (accuracy 96%, AUC 0.99), the relatively small test set (*n* = 94) warrants cautious interpretation. The use of transfer learning and data augmentation may increase the risk of overfitting, particularly in limited datasets. However, the similarity between training and validation loss curves and the absence of divergence during later epochs suggest that substantial overfitting did not occur. Nevertheless, larger external test cohorts are required to confirm model robustness.

However, the study has some limitations. Classification is limited to Zirconia-based fixed restorations and porcelain-fused-to-metal (PFM) restorations, and the generalizability of the model to other types of fixed prostheses remains limited. The dataset was single-center and retrospective, and factors including the number, type, and location of restorations were not comprehensively analyzed. Therefore, the model should be validated using larger and more diverse datasets from multiple clinical centers. The a priori power analysis indicated a minimum of 750 images; however, only 593 original ROI images met the inclusion criteria. While data augmentation improved class balance and model stability, augmented samples do not represent independent biological variability and therefore do not increase true statistical power. For this reason, statistical interpretation should be based on the original dataset size, which constitutes a methodological limitation. Although augmentation techniques were restricted to realistic geometric and photometric transformations and were applied exclusively within the training dataset to prevent data leakage, artificially generated samples cannot fully replace the diversity of independent clinical data. Future studies should prioritize expanding original multicenter datasets rather than relying solely on synthetic balancing approaches.

While this represents a limitation, the use of transfer learning and data augmentation partially compensated for the reduced dataset size. Nevertheless, larger multicenter datasets are required to confirm the robustness and generalizability of the findings. Data augmentation, while necessary to address class imbalance, may have introduced artificial similarity patterns within the zirconia class. Although augmentation techniques were limited to realistic geometric and photometric transformations, the possibility of subtle bias cannot be excluded.

Analysis of the misclassified cases (*n* = 4) revealed that borderline radiopacity patterns and overlapping anatomical structures contributed to classification errors. In some instances, thin metal substructures or variations in restoration thickness reduced contrast differences between material types. These findings highlight that material discrimination on panoramic radiographs may be more challenging in cases with limited radiographic contrast or superimposition artifacts. Such borderline cases provide important insight into the model’s practical limitations and reliability boundaries.

The study was conducted using a single-center dataset acquired with a single imaging device (Planmeca ProMax) and standardized exposure parameters. Variations in scanner models, exposure parameters, image post-processing algorithms, and patient demographic characteristics may influence radiographic contrast and noise patterns. Therefore, the performance of the proposed model across different imaging systems and heterogeneous populations remains uncertain. Future multicenter studies incorporating diverse acquisition protocols are necessary to assess model stability and cross-device generalizability.

Consequently, the model’s generalizability to different imaging systems, acquisition protocols, and patient populations remains untested. External multicenter validation is not merely recommended but is essential before clinical implementation of this model can be considered.

From a clinical perspective, automated material classification may assist clinicians in situations where prior treatment records are unavailable. In routine clinical practice, the proposed model may function as a decision-support tool rather than an autonomous diagnostic system. Potential integration scenarios include preliminary screening during radiographic review, retrospective classification of materials in digital patient archives, and assistance in retreatment planning where restorative material type influences removal strategy or bonding protocol selection. Such integration may enhance workflow efficiency and reduce reliance on incomplete historical documentation.

Differentiating zirconia from metal-supported restorations may influence retreatment decisions, marginal evaluation, removal strategies, and selection of compatible restorative materials.

Clinically, this model may be valuable in scenarios where the restorative material is unknown, such as in patients presenting without prior dental records. It may support decision-making during retreatment planning, removal of existing restorations, and selection of compatible bonding systems. Additionally, automated material classification may facilitate structured digital archiving systems and serve as an educational tool in dental training programs by enabling objective material identification in radiographic interpretation exercises.

By shifting the focus from traditional type-based approaches to material-based classification, this study provides a novel and functional contribution to the development of clinical decision support systems. The high accuracy and F1 values obtained suggest that artificial intelligence may serve as a reliable and specific preliminary diagnostic tool in restorative dental treatments.

Future studies may expand on these findings by incorporating larger and more diverse multicenter datasets, evaluating additional prosthetic materials, and validating models across different imaging devices and acquisition protocols. Furthermore, integrating explainable AI approaches could enhance clinical trust and adoption, while real-time decision-support integration may facilitate broader clinical usability.

## 5. Conclusions

Within the limitations of this single-center retrospective study, the deep learning model demonstrated high performance in differentiating zirconia-based and metal-supported porcelain fixed restorations on panoramic radiographs. These findings suggest that material-based AI classification may contribute to the development of clinical decision-support systems; however, further validation using larger multicenter datasets is required.

## Figures and Tables

**Table 1:** Dataset division.

Dataset	Split (%)	Number of Images
Training	70	648
Validation	20	185
Test	10	94

**Table 2:** Confusion Matrix for the Artificial Intelligence Model.

	Prediction	
	Not	Yes
**Actual**	TN	FP *
	FN *	TP

* Incorrect predictions.

**Table 3:** Class-Based Evaluation (Each Class Accepted as Positive Separately).

		Estimate	
		Metal	Zirconia
**Actual**	Metal	41	2
	Zirconia	2	49

**Table 4:** Precision, Recall, and F1 Score Values Calculated for Each Class.

Class	Precision	Recall	F1 Score	Number of Images
**Metal**	0.95	0.95	0.95	43
**Zirconia**	0.96	0.96	0.96	51

## Data Availability

The datasets generated and/or analyzed during the current study are not publicly available due to institutional restrictions but are available from the corresponding author upon reasonable request.
